# *Clostridioides difficile* strain-dependent and strain-independent adaptations to a microaerobic environment

**DOI:** 10.1099/mgen.0.000738

**Published:** 2021-12-15

**Authors:** Andy Weiss, Christopher A. Lopez, William N. Beavers, Jhoana Rodriguez, Eric P. Skaar

**Affiliations:** ^1^​ Department of Pathology, Microbiology, and Immunology, Vanderbilt University Medical Center, Nashville, TN, USA; ^2^​ Vanderbilt Institute for Infection, Immunology, and Inflammation, Vanderbilt University Medical Center, Nashville, TN, USA; ^3^​ Department of Biological Sciences, California State University Sacramento, Sacramento, CA, USA

**Keywords:** oxygen, *Clostridioides difficile*, RNA-sequencing, bacterial pathogenesis, adaptation to stress

## Abstract

*
Clostridioides difficile* (formerly *Clostridium difficile *) colonizes the gastrointestinal tract following disruption of the microbiota and can initiate a spectrum of clinical manifestations ranging from asymptomatic to life-threatening colitis. Following antibiotic treatment, luminal oxygen concentrations increase, exposing gut microbes to potentially toxic reactive oxygen species. Though typically regarded as a strict anaerobe, *C. difficile* can grow at low oxygen concentrations. How this bacterium adapts to a microaerobic environment and whether those responses to oxygen are conserved amongst strains is not entirely understood. Here, two *C. difficile* strains (630 and CD196) were cultured in 1.5% oxygen and the transcriptional response to long-term oxygen exposure was evaluated via RNA-sequencing. During growth in a microaerobic environment, several genes predicted to protect against oxidative stress were upregulated, including those for rubrerythrins and rubredoxins. Transcription of genes involved in metal homeostasis was also positively correlated with increased oxygen levels and these genes were amongst the most differentially transcribed. To directly compare the transcriptional landscape between *C. difficile* strains, a ‘consensus-genome’ was generated. On the basis of the identified conserved genes, basal transcriptional differences as well as variations in the response to oxygen were evaluated. While several responses were similar between the strains, there were significant differences in the abundance of transcripts involved in amino acid and carbohydrate metabolism. Furthermore, intracellular metal concentrations significantly varied both in an oxygen-dependent and oxygen-independent manner. Overall, these results indicate that *C. difficile* adapts to grow in a low oxygen environment through transcriptional changes, though the specific strategy employed varies between strains.

## Data Summary

The raw sequence data for this study can be found at NCBI GEO, accession no. GSE173804. Analysed data can be found in Tables S1–S5 (available in the online version of this article). The authors confirm all supporting data and protocols have been provided within the article or through supplementary data files.

**Impact Statement**

*
Clostridioides difficile* is considered a strict anaerobe and is routinely cultured in an environment free of oxygen. Yet, there is an evolving understanding that *C. difficile* encounters oxygen during colonization of the gut and subsequently during toxin-mediated inflammation. Furthermore, the genetic and regulatory basis of phenotypic heterogeneity amongst *C. difficile* strains is incompletely known but probably contributes to complications in clinical pathogen detection and patient treatment. In this study, we used RNA-sequencing to elucidate the large-scale transcriptional changes that occur during growth in a microaerobic environment in two *C. difficile* strains. Our data reveal that *C. difficile* can adapt to grow in an oxygenated environment in part through differential expression of numerous genes, including those involved in mitigating oxidative stress and maintaining metal homeostasis. Furthermore, we directly compared expression patterns between *C. difficile* strains under anaerobic and microaerobic conditions by creating a consensus genome. The oxygen-dependent and -independent variability in gene expression between strains in this study suggests caution should be taken when interpreting *C. difficile* transcriptional responses to environmental stimuli from single strains.

## Introduction

*Clostridioides difficile* is a Gram-positive, spore-forming bacterium found in the intestines of a diverse range of mammals [1, 2]. In humans, ingestion of *C. difficile* spores and disruption of the commensal microbiota, typically through antibiotic use, results in a *C. difficile* bloom [3]. Following spore germination, these vegetative *C. difficile* cells produce toxins that mediate disease progression [4]. *C. difficile* infection (CDI) can present as a spectrum of symptoms, from mild diarrhoea to pseudomembranous colitis and toxic megacolon, which can eventually lead to death [5]. Yet, *C. difficile* can also colonize the gut asymptomatically and in the absence of known risk factors [2, 6, 7]. This ability to colonize the gut under a variety of conditions, from homeostatic to inflamed, is one of the pathogen’s defining features [2].

*C. difficile* is considered an obligate anaerobe that thrives in a gut environment that under homeostatic conditions is maintained as mostly anoxic through the combined actions of the microbiota and the host [8]. Under homeostatic conditions, the short-chain fatty acid butyrate is produced by the gut microbiota and serves as an energy source for colonic epithelial cells, or colonocytes [8, 9]. Butyrate acts on the peroxisome proliferator activator receptor gamma (PPARγ) in differentiated colonocytes to drive beta-oxidation for energy production [9, 10]. Beta-oxidation requires oxygen; thus, colonocyte metabolism consumes oxygen that would otherwise diffuse into the intestinal lumen and keeps luminal oxygen tensions low (<10 mmHg) [11]. Antibiotic treatment increases luminal oxygenation by reducing populations of butyrate-producing members of the microbiota [12]. Reduced butyrate concentrations and lower PPARγ activation shift colonocyte metabolism towards oxidative fermentation, leaving oxygen to freely diffuse across the colonocyte apical surface and become available in the gut [8]. In accordance with this connection between the microbiota, colonocyte metabolism, and luminal oxygen availability, mice rendered susceptible to CDI through antibiotic administration have decreased levels of short-chain fatty acids [13] and increased tissue oxygenation [14]. Furthermore, mice provided butyrate following CDI have decreased epithelial oxygenation [15], suggesting that butyrate availability is a driving factor in controlling oxygen tensions in the gut. Overall, these studies reveal that *C. difficile* probably encounters low levels of oxygen that stem from dysbiosis of the gut microbiota and changes in host metabolism.

Rapid exposure to atmospheric oxygen levels initiates a shock response that is presumably aimed at providing short-term protection to *C. difficile* vegetative cells [16]. Exposure to low oxygen levels (up to 5 %) for limited periods (15 and 60 min) causes *C. difficile* to alter transcription of genes involved in amino acid fermentation, carbohydrate metabolism and cofactor biosynthesis [17]. Additionally, oxygen exposure increases *C. difficile* protein oxidation and induces transcription of an oxidative stress response programme to repair oxidized proteins and reduce molecular oxygen to water [17, 18]. While these studies demonstrate that *C. difficile* has evolved mechanisms to survive short periods of high oxygen, with 2% oxygen *C. difficile* growth is severely limited [19] and at higher concentrations vegetative cells quickly lose viability [16]. Despite encountering oxygen in the gut, *C. difficile* is able to bloom and initiate disease, suggesting that oxygen levels in the gut are below growth-inhibitory concentrations. The adaptations required for *C. difficile* to grow at physiologically relevant levels of oxygen for extended periods of time are not clear.

In this study, we analysed how *C. difficile* strain 630 (ribotype 012) [2] and an historical CD196 strain (ribotype 027) [20] adapt to an environment of 1.5% oxygen. Using an initial RNA-sequencing (RNA-seq) approach, we found that under microaerobic conditions both *C. difficile* strains increased transcription of genes predicted to protect against oxidative stress while altering transcription of carbohydrate and amino acid fermentation genes. Interestingly, microaerobic growth also induced a transcriptional response similar to a nutrient metal starvation response, with concomitant changes in intracellular metal concentrations. While many of the observed responses to oxygen were conserved between the two *C. difficile* strains, we also observed oxygen-dependent and oxygen-independent differences. A cross-comparison of transcript abundance based on a ‘consensus genome’ and follow-up experiments revealed strain-dependent differences in amino acid metabolism and intracellular metals. Together, these data reveal that *C. difficile* adapts to physiologically relevant oxygen concentrations through transcriptional shifts in metabolic genes that can be strain-specific.

## Methods

### Bacterial strains and culture conditions

*C. difficile* strain CD196 (ribotype 027) [20] and strain 630 (ribotype 012) [2] were routinely cultured in brain-heart infusion media supplemented with yeast extract (BHIS) (52 g l^−1^ BHI, 5 g l^−1^ yeast extract, 0.03% l-cysteine) [21]. Routine cultivation of *C. difficile* was performed in an atmosphere of 5% CO_2_, 5 % H_2_ and 90 % N_2_ in an anaerobic chamber (Coy). To test the effects of oxygen, *C. difficile* cultures were moved to a hypoxia chamber (Coy) set at 1.5% oxygen, with the remaining gas being CO_2_, H_2_ and N_2_ (1:1:18 ratio, as above).

### RNA extraction

*C. difficile* strains were first cultured under anaerobic conditions overnight in BHIS. Cultures were then diluted 1:50 into 5 ml of BHIS broth (pre-conditioned either under anaerobic conditions or with 1.5% oxygen) in each well of a six-well plate. Plates were then incubated under aerobic or anaerobic conditions at 37 °C until bacteria reached the mid-exponential phase, which was evaluated by measuring the optical density at 600 nm (OD_600_) using a 1 ml cuvette. The average OD_600_ and time to reach this optical density for each strain and condition were: CD196 anaerobic (0.333, 4.5 h); CD196 microaerobic (0.440, 5 h); 630 anaerobic (0.406, 6.5 h); 630 microaerobic (0.370, 8 h). When cultures reached the desired OD_600_, media from two wells of the same bacteria and same condition were transferred to a 15 ml conical tube and centrifuged to pellet bacteria. Supernatant was removed and pellets were used for RNA extraction. RNA for RNA-seq experiments was extracted from samples grown in biological triplicate.

To extract RNA, samples were maintained on ice. Pellets were first resuspended in 1 ml TriReagent, then transferred to lysing matrix B tubes. Bacteria were lysed using a bead beater set at 6 m s^–1^ for 50 s. Lysate was left at room temperature for 5 min, then spun down at 13000 g for 1 min. The TriReagent–lysate mixture was then transferred to a new tube. The homogenate was mixed with 200 µl chloroform and incubated at room temperature for 5 min, followed by centrifugation at 12000 g for 15 min at 4 °C. The aqueous phase only was next transferred to a new tube and mixed with 500 µl isopropanol. Following precipitation, RNA was pelleted by centrifugation at 12000 g for 8 min at 4 °C. The RNA pellet was washed with 1 ml of 75% ethanol and then air dried. RNA was then suspended in water. Contaminating DNA was removed using the TURBO DNA-free kit (Invitrogen).

### Sample preparation and RNA-seq

RNA quantity was determined using a Qubit Fluorometer (Thermofisher) and RNA quality was assessed through the use of a Bioanalyzer TapeStation with the RNA 6000 Nano kit (both Agilent). rRNA was depleted by application of the RiboMinus Transcriptome Isolation kit (bacteria) and the RiboMinus Concentration Module (both Invitrogen). Library construction was performed using the NEBNext Ultra II RNA Library Prep Kit for Illumina and NEBNext Multiplex Oligos for Illumina (both NEB). Sequencing of the constructed libraries was performed using a NovaSeq 6000 instrument in combination with the NovaSeq 6000 S4 Reagent Kit (both Illumina). The described workflow resulted in an average of 58 million reads per sample. All generated sequencing data were deposited at the NCBI GEO (accession no. GSE173804).

### Data analysis

Unless noted otherwise, all bioinformatic analyses were performed using CLC Genomics Workbench (version 20.0.1.). Paired-end sequencing data in fastq format were imported into CLC Genomics Workbench and trimmed to remove barcodes and adapter sequences. To computationally deplete remaining rRNA reads, the entirety of the generated reads were aligned to a sequence list containing all rRNA sequences for the respective *C. difficile* genomes. Unmapped (non-rRNA) reads were collected and utilized for subsequent analysis. Reference genomes NC_009089 and NC_013315 were used for 630 and CD196, respectively. For intra-strain comparison, tracks were generated for each genome, before employing the ‘RNA-Seq Analysis’ function (standard settings, similarity fraction of 0.8) to generate expression values for each gene. Comparison of gene expression patterns under anaerobic and microaerobic conditions was performed using the ‘Differential Expression in Two Groups’ function.

For cross-comparison between the two strains in this study, a ‘consensus genome’ was generated and used instead of individual reference genomes. The ‘consensus genome’ was created by performing a blast search of every 630 gene against each gene in the CD196 genome using the ‘MultiBLAST’ function in CLC Genomics Workbench (standard settings). For each individual blast event, the top hit was determined, and the consensus sequence was extracted (80% identity cutoff with manual curation in the 80–90% range; 1E-70 *P*-value cutoff; insertion of ‘N’ ambiguity symbols for conflicts). The resulting list of 3344 consensus genes was then converted into tracks and used for subsequent RNA-seq analysis and comparison as described for intra-strain comparisons described above.

### Data visualization

Volcano pots and distribution maps denoting fold-changes across the genome (see Fig. 2) were generated using GraphPad Prism and on basis of fold-changes and false discovery rate (FDR) *P*-values calculated by CLC Genomics Workbench. A whole genome alignment was created using the ‘Whole Genome Alignment (beta)’ function in CLC Genomics Workbench.

### Quantitative PCR

To quantify RNA, *C. difficile* was cultured and RNA was extracted as above (see ‘RNA extraction’). In total, 1.5 µg of RNA was reverse transcribed by M-MLV reverse transcriptase (Fisher Scientific) in the presence of RNase inhibitor (Promega), dNTPs (Promega) and random hexamers (Promega). Newly synthesized cDNA was diluted 1:200 in water and used in quantitative reverse transcriptase PCR (qRT-PCR) using iQ SYBR green supermix (BioRad). Amplification was achieved using a three-step melt curve programme on a CFX96 qPCR cycler (BioRad). Target gene expression using the primers listed in (Table 1) was normalized to the *rpoB* housekeeping gene using the ΔΔCt method.

**Table 1.** Oligonucleotides used in this study

Primer name	Sequence (5′−3′)	Reference
*rbo*-qPCR-FW	AGTAAGTAGCACAACTCATCCA	This study
*rbo*-qPCR-RV	CAGTAAGCATAGGCAGAAATCA	This study
*rpoB*-qPCR-FW	TGCTGTTGAAATGGTTCCTG	[64]
*rpoB*-qPCR-RV	CGGTTGGCATCATCATTTTC	[64]
*rbr-1*-qPCR FW	GCAGGAGAGTCAGAAGCAAG	This study
*rbr-1*-qPCR RV	TTCACCTGCTGCTGCATC	This study
*CD630_08260*-qPCR FW	AAATATTAAATAATCCGGTTCATCCTAC	This study
*CD630_08260*-qPCR RV	TCGTATACCTCTCCACATACTTC	This study
*trxA_2*-qPCR FW	TCTCCTTAGGCATAAAACCTATTAATC	This study
*trxA_2*-qPCR RV	TTGCGACTTGGTGTGGAC	This study
*CD630_15070*-qPCR FW	AGATACGGAGGATGTTGATGG	This study
*CD630_15070*-qPCR RV	TTCAAGCTCTGGCATTTCAC	This study
*fhuB*-qPCR FW	TTGCAGGAGTCGCTCTTGGT	This study
*fhuB*-qPCR RV	TCACTCCTACAAGCCCTCCTGA	This study
*feoA1*-qPCR FW	AGAAGGAGAAGGAGCTACAC	This study
*feoA1*-qPCR RV	TCTGCATCTGACTTACGAATTG	This study
*feoA2*-qPCR FW	GTCTTACTAGAGGAGCTGTTATAG	This study
*feoA2*-qPCR RV	ACTTTCTTCTTGTCGTAGAGC	This study
*prdA*-qPCR FW	GGTCAAGTACTAGGAGCTAAGT	[23]
*prdA*-qPCR RV	CTACTTCTTCTTTAGCCTCTCCTG	[23]
*grdE* FW	CCCTGGTATCATGTCTAAAGTTG	[44]
*grdE* RV	GAGTATACTTAGCTCCTTCTCCAG	[44]

### Animal infection

#### Mouse model of CDI

Age-matched adult (7–10 weeks old) male C57BL/6J mice were purchased from Jackson Labs. The cefoperazone mouse model of CDI used in this study follows the protocol previously described [22]. Briefly, mice were treated with 0.5 mg ml^−1^ cefoperazone in their drinking water for 5 days, followed by 48 h of recovery on normal drinking water. For infections, mice were inoculated via oral gavage with 1×10^5^ spores in 100 µl of water of either *C. difficile* CD196 or 630. Prior to infection, mice were confirmed to be *C. difficile *-negative via plating on taurocholate cycloserine cefoxitin fructose agar (TCCFA). *C. difficile* causes inflammation of the colon, and thus colon tissue samples were collected from mice on day 0 (mock, no *C. difficile *) or days 1, 2 and 3 post-inoculation. Prior to sample collection, mice were given 100 µl of PBS containing 20 mg ml^−1^ pimonidazole HCl (PMDZ) via intraperitoneal injection. After 1 h, mice were killed and approximately 2 cm colon sections were preserved in 10% formalin solution. Colon samples were then paraffin-embedded and sectioned for staining.

To determine bacterial burdens, colon contents were weighed and homogenized in PBS and serially diluted then plated onto TCCFA for enumeration as colony-forming units (c.f.u.) per gram of faeces.

### Hypoxia staining

To detect hypoxia in PMDZ-treated colon samples, sectioned samples on slides were first deparaffinated. Antigen retrieval was performed by covering samples with a proteinase K solution (20 µg ml^−1^ in TE buffer) and permeabilized with a 0.2% Tween-20 solution in PBS. Staining was performed with a primary mouse anti-PMDZ antibody (MAb1 4.3.11.3 Lot #9.7.11 Hydroxyprobe) and a secondary goat anti-mouse AlexaFluor 555 antibody. Tissues were counterstained with Hoechst (1 µg ml^−1^) and mounted using ProLong gold antifade.

### Growth curves

Costar 96-well strip plates were filled with 196 µl per well of BHIS and then placed into either an anaerobic chamber or a hypoxia chamber set at 1.5% oxygen to determine *C. difficile* growth under anaerobic or aerobic conditions, respectively. After 24 h of preconditioning, overnight cultures of *C. difficile* strains 630 and CD196 were diluted 1:50 into the wells (*n*=4 per strain per condition). Inoculated plates were then immediately returned to the anaerobic chamber or hypoxia chamber. At each time point, one strip from each plate under each oxygen condition, which consisted of both CD196- and 630-inoculated wells, was removed and the OD_600_ was measured. These strips were then discarded while the remaining strips from each plate remained in the appropriate oxygen condition to continue incubating.

Growth curves were monitored up to 11 h post-inoculation. To determine growth after 16 h of incubation, 5 ml of BHIS broth in each well of a six-well plate was pre-conditioned either in an anaerobic chamber or a hypoxia chamber set at 1.5, 2 or 5% oxygen. Wells were then inoculated with a 1:50 dilution of overnight *C. difficile* 630 or CD196 cultures and incubated under either anaerobic or aerobic conditions at 37 °C. After 16 h of incubation, medium was diluted with PBS, and *C. difficile* vegetative cells were enumerated by plating on BHIS agar plates anaerobically.

### Survival assay

To determine the survival of vegetative *C. difficile* over a short period, 5 ml of BHIS broth in each well of three six-well plates was pre-conditioned either in an anaerobic chamber, at atmospheric oxygen conditions (21 %, or a hypoxia chamber set at 1.5% oxygen. Overnight cultures of *C. difficile* 630 and CD196 were diluted 1:100 in fresh BHIS media and further incubated for 6 h at 37 °C anaerobically. The sub-cultured *C. difficile* was used to inoculate the six-well plates under the different conditions. At timepoint 0, 10, 30 and 60 min, a sample from each well under all conditions was diluted in PBS and then plated on BHIS agar anaerobically.

### Inductively coupled plasma MS (ICP-MS)

To quantify intracellular metal concentrations, 5 ml of BHIS broth in each well of two six-well plates was pre-conditioned either in an anaerobic chamber or a hypoxia chamber set at 1.5% oxygen. Wells were then inoculated with a 1:50 dilution of overnight *C. difficile* 630 or CD196 cultures and incubated under either anaerobic or aerobic conditions at 37 °C. Once the OD_600_ reached approximately 0.330, 1 ml of culture was transferred to a pre-weighed metal-free tube. Samples were centrifuged at high speed and the supernatant was removed. Cells were washed with distilled water. After washing, tubes were weighed again to determine the weight of the bacterial pellet. To prepare for ICP-MS, bacterial pellets were incubated at 65 °C overnight in 200 µl Optima nitric acid and 50 µl 30% hydrogen peroxide. Following acid and hydrogen peroxide digestion, 1 ml of UltraPure water was added.

ICP-MS was performed as described previously [23]. Elemental quantification was performed using an Agilent 7700 ICP-MS (Agilent) attached to a Teledyne CETAC Technologies ASX-560 autosampler (Teledyne CETAC Technologies). The following settings were fixed for the analysis: cell entrance = −40 V; cell exit=−60 V; plate bias=−60 V; octP bias=−18 V; collision cell helium flow=4.5 ml min^−1^. Optimal voltages for Extract 2, Omega Bias, Omega Lens, OctP RF and Deflect were determined empirically before each sample set was analysed. Element calibration curves were generated using ARISTAR ICP standard mix (VWR). Samples were introduced by a peristaltic pump with 0.5 mm internal diameter tubing through a MicroMist borosilicate glass nebulizer (Agilent). Samples were initially taken up at 0.5 r.p.s. for 30 s followed by 30 s at 0.1 r.p.s. to stabilize the signal. Samples were analysed in Spectrum mode at 0.1 r.p.s. collecting three points across each peak and performing three replicates of 100 sweeps for each element analysed. Sampling probe and tubing were rinsed for 20 s at 0.5 r.p.s. with 2% nitric acid between every sample. Data were acquired and analysed using the Agilent Mass Hunter Workstation Software version A.01.02. Elemental data were normalized to the concentration of sulphur.

### 5-Aminovalerate measurements

Overnight cultures of *C. difficile* were used to inoculate 5 ml of pre-conditioned BHIS broth (pre-conditioned in an anaerobic chamber) in six-well plates. These were then incubated at 37 °C until bacteria reached the early exponential phase (3.5 h post-inoculation). The OD_630_ was recorded for later normalization and 600 µl of culture was then transferred to an Eppendorf tube. Samples were centrifuged at a high speed and supernatant was then transferred to a 0.22 µm filter tube. After centrifugation, the flow-through was collected for downstream processing.

5-Aminovalerate was measured from samples using methods described previously [23]. Heptafluorobutyric acid (HFBA; Sigma) was added to each sample to 75 mM as an ion pairing agent. Samples were analysed on a Thermo TSQ Quantum Ultra with an ESI source interfaced to a Waters Acquity UPLC system. Analytes were separated by gradient HPLC with an Agilent Poroshell 120 C_18_ (3.0×50 mm, 2.7 µm) and a Phenomenex SecurityGuard C_18_ cartridge (3.2×8 mm) at a flow rate of 0.3 ml min^−1^ using 10 mM HFBA in water and 10 mM HFBA in acetonitrile as mobile phases A and B, respectively. The gradient was held at 0% B for 1 min, then ramped to 100% B over the next 8 min. The column was washed at 100% B for 3 min, then equilibrated to 0% B for 3 min. 5-Aminovalerate was analysed by multiple reaction monitoring in negative ionization mode at an *m*/*z* transition for 5-aminovalerate (118.1 to 55.1) using a collision energy of 16 eV. Skimmer offset and tube lens voltages were determined empirically before each set of samples was run. Quantification was performed by comparing analyte AUC (area under the curve) values to those of an external calibration line of 5-aminovalerate at 0, 1, 3, 10, 30, 100, 300 and 1000 µM.

## Results and Discussion

### Colon tissue oxygenation increases following antibiotic treatment and during CDI

**Fig. 1.**
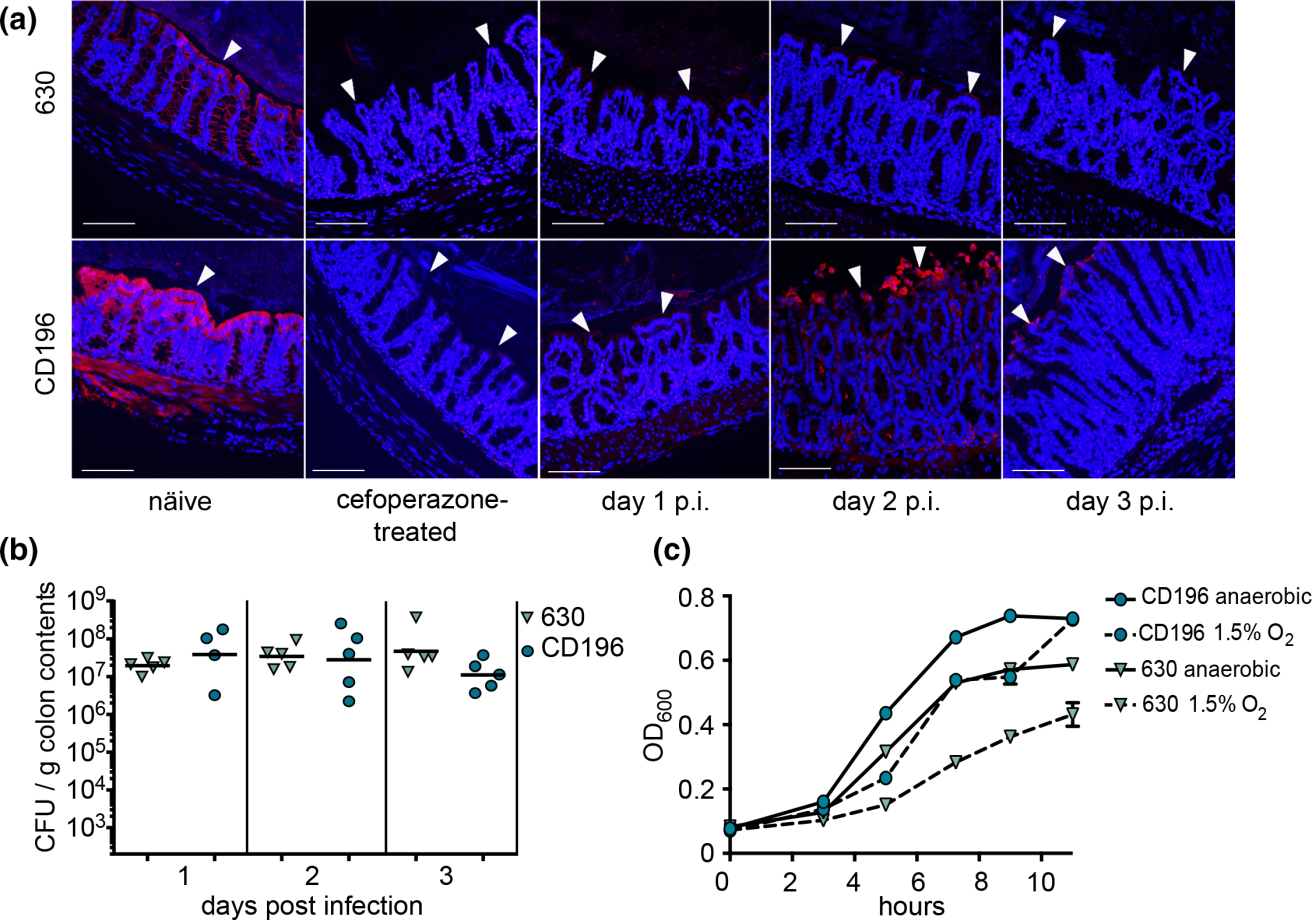
Colonic epithelial cells increase oxygenation during infection with *C. difficile* strains 630 and CD196. (**a**) Mouse colon sections of untreated naïve, cefoperazone only-treated and cefoperozone-treated and infected mice up to 3 days post-infection (p.i.) stained for the hypoxia marker PMDZ. Cell nuclei are shown as blue (Hoechst) and PMDZ staining is shown as red. Arrowheads indicate the apical surface of colonic epithelial cells. Bar: 100 µm. Each image is representative of the colon section for an individual mouse, with none of the mice repeated in the figure. (**b**) *C. difficile* burdens of mice shown in (**a**) infected with either strain 630 or CD196. (**c**) Growth of *C. difficile* strains 630 and CD196 in BHIS broth under anaerobic conditions or in 1.5% oxygen (*n*=4).

Antibiotic treatment and disruption of the microbiota, both significant risk factors for developing CDI, influence colonic tissue oxygen tensions [12]. To determine how tissue oxygenation changes during CDI, mice were treated with the broad-spectrum antibiotic cefoperazone and subsequently inoculated with spores of *C. difficile* strain 630 [2]. Colon tissue sections were then collected from uninfected and infected mice and stained for the hypoxia marker pimonidazole (PMDZ). PMDZ binds to protein thiols when oxygen tensions fall below approximately 1.2% (10 mmHg), while increasing oxygen levels prevent PMDZ binding [24]. In naïve mice that received neither antibiotics nor spores, the differentiated colonocytes near the intestinal lumen stained highly for PMDZ (Fig. 1a), indicating that these cells were hypoxic. Five days after antibiotic treatment, PMDZ staining was no longer evident. This increased tissue oxygenation persisted for up to 3 days post-infection during which time *C. difficile* bloomed to reach burdens of roughly 10^7^ c.f.u. per gram of faeces (Fig. 1a, b). These results show an increase in tissue oxygenation following antibiotic treatment and subsequent infection with *C. difficile* 630 and are consistent with a previous report [14].

*C. difficile* strains are genetically diverse due to a high rate of genome rearrangement [20, 25] and commonly have variable phenotypes clinically and in culture [26–30]. To determine whether increased tissue oxygenation is a common feature amongst strains during CDI, mice were infected with a *C. difficile* ribotype 027 strain, CD196. *C. difficile* CD196 reached similar levels of gut colonization as strain 630 (Fig. 1b) and at day 1 post-infection tissue hypoxia levels were similar between the two strains (Fig. 1a). *C. difficile* CD196 induces more severe disease in mice than strain 630 [22] and by day 2 post-infection PMDZ-stained hypoxic cells can be visualized sloughing off at the lumen–colonocyte interface. However, colonocytes attached to the epithelium exhibit decreased PMDZ staining compared to naïve mice on both days 2 and 3 post-infection (Fig. 1a), indicating that increased tissue oxygenation is a common feature during infection by these strains. Studies examining the responses of pathogenic *Enterobacteriaceae* to oxygen in the inflamed intestines revealed that this increased tissue oxygenation leads to low levels of oxygen in the lumen that can be respired by facultative anaerobes [12, 31]. Therefore, it is reasonable to suggest that *C. difficile* is exposed to oxygen early during infection.

### Microaerobic conditions induce global changes in transcription in *C. difficile* strains 630 and CD196

Our results showed that following antibiotic treatment and during CDI there is a decrease in PMDZ intracellular staining. Since PMDZ binding is prevented by limited oxygen, the loss of PMDZ staining indicates oxygen tensions above approximately 1.2% [24] (Fig. 1a). *C. difficile* fails to grow at 5% oxygen *in vitro* [19], but blooms in the intestines within the first days of initial infection (Fig. 1b). Thus, we reasoned that an oxygen concentration of 1.5% is physiologically relevant and would permit growth of *C. difficile* to allow an assessment of how *C. difficile* adapts to grow in the presence of oxygen. This rationale was supported by growing *C. difficile* strains 630 and CD196 in culture for 16 h at 0, 1.5, 2.0 and 5.0% oxygen in BHIS, but without cysteine supplementation. Both strains grew to high abundance at 0 and 1.5% oxygen, but failed to grow at 2 and 5% oxygen (Fig. S1). On a shorter timescale, within 1 h at 2 % oxygen vegetative cells quickly lost viability (Fig. S2). Since *C. difficile* failed to thrive at 2% oxygen, but grew at 1.5% oxygen, we continued to measure the growth of *C. difficile* strains 630 and CD196 anaerobically and in the presence of 1.5% oxygen (Fig. 1C). Interestingly, there was a noticeable growth difference between the strains under both conditions, with CD196 growing at a faster rate during the exponential phase than strain 630 (Fig. 1c). At 0 and 1.5% oxygen, both strains reached high levels after 16 h, though c.f.u. counts were slightly higher for CD196 (Fig. S1). These distinct growth dynamics indicate underlying genetic variability that could in part explain the phenotypic heterogeneity amongst *C. difficile* strains.

**Fig. 2.**
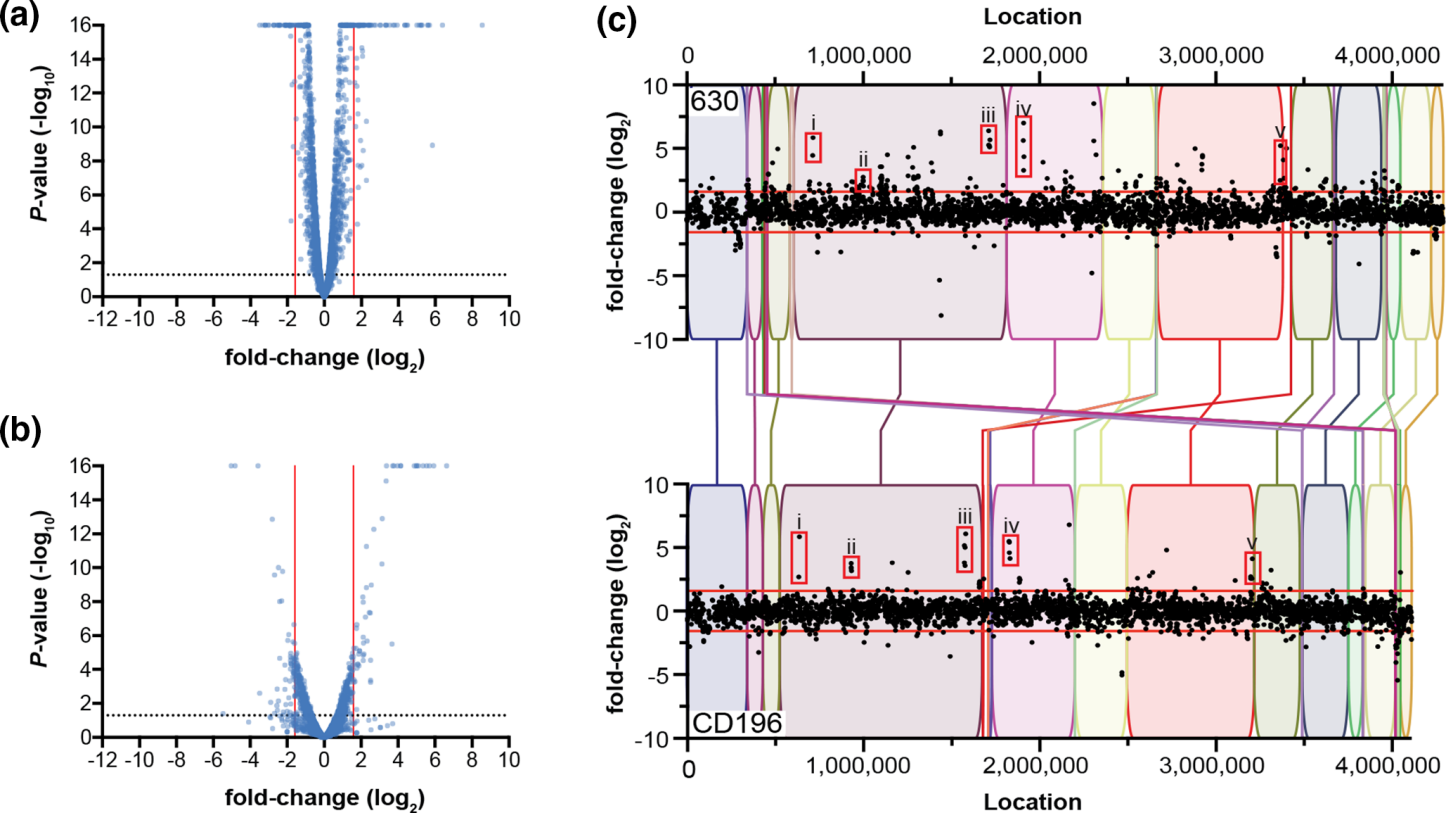
*
C. difficile* reacts to environmental oxygen through large-scale alterations in gene expression patterns. RNA-seq of *C. difficile* grown anaerobically or in the presence of 1.5% oxygen was performed. For strains 630 and CD196, growth in microaerobic conditions leads to changes in the expression of various genes when compared to anaerobically grown cultures. (**a, b**) Volcano plots showing log_2_ fold-change in expression under microaerobic conditions for strains 630 (**a**) and CD196 (**b**). (**c**) Fold-changes, both statistically significant and non-significant, are shown as black dots, with red lines highlighting fold-changes of ±3. The boxes in each genome highlight homologous gene clusters for (i) heavy-metal transporters, (ii) oxidative stress response genes *rbr*, *rbo* and *perR*, (iii) iron transporters *feoA1B1*, (iv) iron ABC transport cluster and (v) a putative siderophore transporter system.

Previous studies largely focused on the short-term *C. difficile* oxidative shock response [16, 17]. However, many bacteria considered to be strict anaerobes, such as *Clostridia *, can tolerate and grow in oxic environments [32]. We hypothesized that exposure to low oxygen over time initiates a transcriptional response that is distinct from rapid exposure to high concentrations of oxygen. To determine the genes important in the *C. difficile* adaptation to microaerobic growth, RNA-seq was performed on RNA isolated from 630 and CD196 at mid-exponential phase cultured under anaerobic and microaerobic (1.5% oxygen) conditions (Table S1). Both strains exhibited large-scale transcriptional changes in response to oxygen (Fig. 2, Table S2). Of note, we observed slight differences in statistical confidence for transcriptional changes in both strains (Fig. 2a, b), which is probably the result of variable success during rRNA removal for individual samples. We account for this difference by applying strict cutoff criteria for downstream analysis (*P*<0.05, fold-change >3). Under microaerobic conditions, 212 genes from *C. difficile* 630 were differentially expressed with fold-changes greater than 3 (Fig. 2a, Table S2). *C. difficile* CD196 overall had fewer genes, 99, differentially expressed in microaerobic conditions (Fig. 2b, Table S2). Of the genes that displayed oxygen-dependent expression patterns, the majority were increased in expression for 630 and CD196 (76 and 52.5%, respectively). These findings highlight a widespread transcriptional response to alterations in environmental oxygen in both strains.

To account for the extensive genomic plasticity observed across *C. difficile* isolates, which includes large-scale rearrangements [25], we performed a whole genome alignment of the *C. difficile* 630 and CD196 genomes. Overall, large genomic regions were conserved between strains (Fig. 2c, coloured boxes), though there was evidence of rearrangements in the relative positions of those conserved regions. The alignment was next overlaid with data to map expression changes for every gene (no cut-offs for significance and fold-change were applied) across the entire genome of each strain. This provided a visual assessment of the transcriptional adaptation to increasing oxygen between strains and allowed for a direct comparison of expression changes independent of relative localization of a specific region within each strain’s genome.

Based on this analysis, several genomic loci that displayed similar adaptation patterns were identified in both strains (Fig. 2c, red boxes). These included a locus encoding oxygen detoxification genes (rubrerythrin, *rbr*; rubredoxin oxidoreductase, *rbo*) (Fig. 2c, Box ii), which increased expression under microaerobic conditions relative to expression under anaerobiosis. Induced expression of these genes was expected based on earlier studies [17, 33] and validates the environmental manipulations. Surprisingly, several gene clusters involved in metal homeostasis were also highly upregulated under microaerobic conditions. Prior studies evaluating *C. difficile* responses to short exposures of moderate oxygen concentrations (5% and higher) did not report changes in metal homeostasis, through either gene transcription or protein production [17]. Here, multiple nutrient metal importers (*feoA* and *feoB*; *zupT*) (Fig. 2c, Boxes i, iii, iv) and predicted siderophore transporters (*fhu* operon) (Fig. 2c, Box v) were amongst the most highly upregulated genes.

### Low oxygen affects expression of *C. difficile* genes involved in the response to oxidative stress

**Fig. 3.**
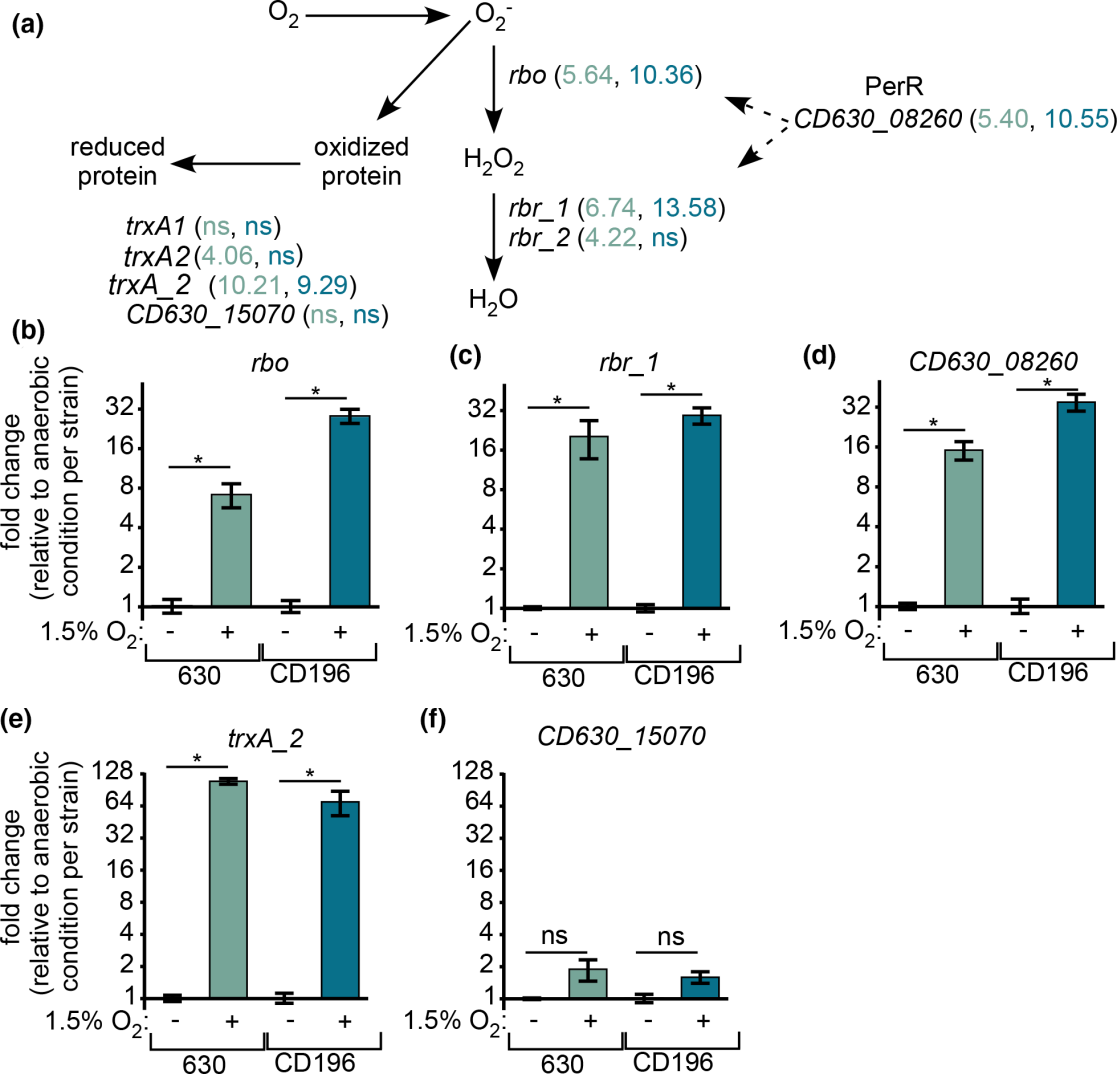
*
C. difficile* increases transcription of oxidative stress genes in a low oxygen environment. (**a**) Outline of genes found in both strains that respond to oxidative stress. Relative fold-change expression values from RNA-seq with 1.5% oxygen are shown for strain 630 (green) and CD196 (blue). (**b–f**) Transcript quantification via qPCR from genes that showed significantly different expression with 1.5% oxygen in the RNA-seq experiment. Values displayed are fold-change with 1.5% oxygen relative to anaerobic conditions for each strain. *P<0.05; ns, not significant; two-tailed *t*-test.

Exposure to oxygen generates reactive oxygen species (ROS) that can inhibit or kill susceptible bacteria. Bacteria that grow in aerobic environments adapt to oxygen stresses by producing protective enzymes that convert ROS to less harmful molecules [34, 35]. A common example in obligate aerobes is the conversion of superoxide radicals to hydrogen peroxide by superoxide dismutase (SOD) followed by conversion of hydrogen peroxide to water and molecular oxygen by catalase. Rubrerythrin peroxidases and superoxide reductases similarly detoxify ROS in many anaerobic bacteria [32]. While *C. difficile* is traditionally cultured under strictly anaerobic conditions, RNA-seq of *C. difficile* grown with 1.5% oxygen revealed upregulation of several genes that produce factors involved in ROS detoxification. For instance, both 630 and CD196 increased expression of *rbo*, a gene encoding a homologue of desulphoferredoxin in *Desulfovibrio vulgarus* that has superoxide reductase activity [36] (Fig. 3a). qPCR of the *rbo* genes corroborated the RNA-seq results, showing that 1.5% oxygen led to significant transcriptional upregulation in 630 and CD196 (Fig. 3b). Further detoxification of hydrogen peroxide is predicted to be accomplished by rubrerythrin enzymes [33, 36]. In response to oxygen, genes encoding two rubrerythrins (*CD630_08250*, *rbr_1*; *CD630_28540*, *rbr_2*) increased expression in 630 while only one (*CD196_RS14495*, *rbr_1*) was significantly upregulated in CD196. Significant upregulation of *rbr_1* was confirmed via qPCR (Fig. 3c). The *rbo* and *rbr_1* genes are upstream and downstream, respectively, of a gene encoding a Fur-like transcriptional repressor that regulates the expression of these surrounding genes [37], and probably plays a similar role in the response to ROS as PerR in other organisms [38]. Expression of this PerR homologue (*CD630_08260*; *CD196_RS04465*) significantly increased with oxygen (Fig. 3a, d, Table S2). Of note, a widely studied erythromycin-sensitive derivative of the parent 630 strain, 630*∆erm*, carries a T41A mutation that leads to constitutive derepression of the PerR regulon [37]. Sequencing of the *perR* loci in both strains used in this study did not show this mutation (data not shown). Given the role of the PerR regulon in a microaerobic environment, the genetic variability between *C. difficile* strains needs to be considered.

Aside from PerR, σ^B^ also contributes to the *C. difficile* oxidative stress response [39, 40]. To determine whether genes in our dataset are also regulated by σ^B^, the published σ^B^ regulon in strain 630*∆erm* was compared to genes differentially expressed with oxygen in our dataset [40]. The σ^B^ regulon appeared to be largely unaffected by changing oxygen availabilities. For strain 630 only, we detected decreased expression of the σ^B^ -controlled flagellin biosynthesis cluster. The minimal overlap supports the notion of complex transcriptional patterns in the response to oxygen that, based on comparisons with related studies, could be influenced by strain genetics, oxygen concentration, and length of oxygen exposure.

Besides Rbo and Rbr, other proteins contribute to *C. difficile* oxygen resistance, including two reverse rubrerythrins (revRbr) [41] and two flavodiiron proteins (FDPs) [33]. These act as NADH-dependent oxygen reductases and enable optimal growth at oxygen concentrations as low as 0.4 and 0.1% [33]. Surprisingly, none of these four genes had altered expression in either 630 or CD196 under the conditions tested. Similarly, we did not observe changes to expression of the gene encoding cysteine desulfurase IscS2, which contributes to *C. difficile* growth *in vitro* in the presence of oxygen [14]. We also did not observe changes in expression of the *C. difficile* HsmRA system [42]. During infection, host haem that is released into the intestinal lumen is sensed by HsmR, which increases expression of *hsmA*. HsmA then incorporates haem to limit oxidative stress [42]. While relevant to the inflammatory conditions during CDI when haem is present, in this study the *hsmRA* genes did not increase expression under microaerobic conditions alone.

When exposed to oxygen, ROS damage *C. difficile* cells in part through uncontrolled protein oxidation [17]. Thioredoxin and thioredoxin reductases mitigate protein oxidation through electron transfer [43]. Both *C. difficile* strains studied here encode multiple copies of thioredoxin genes, some of which may be involved in repairing protein oxidation (e.g. 630 *trxA2 – CD630*_*23550*, *CD196_RS11940*; *trxA_2 CD630_30330*, *CD196_RS15155*). Other thioredoxins are involved in glycine fermentation (*trxB3 - CD630_23560*, *CD196_RS11945,* Fig. S3a) [44, 45]. Several *trx* genes were upregulated in both strains when grown with 1.5% oxygen. The highest expressed thioredoxin in the RNA-seq dataset, *trxA_2*, was validated via qPCR (Fig. 3e). The thioredoxins found in this study differ from those uncovered by a prior transcriptomic analysis of *C. difficile* 630*∆erm* that was exposed to 5% oxygen [17]. In that study, the expression of two thioredoxins (homologous to *CD630_16900* and *CD630_15070* in strain 630) was significantly altered while, notably, expression levels of the thioredoxins *trxA2* and *trxA_2* were not significantly different [17]. To confirm our results that some thioredoxins were not differentially expressed in the tested conditions, qPCR was performed on *CD630_15070*. There were no significant differences in transcript levels between anaerobic and 1.5% oxygen conditions (Fig. 3f).

The variability in oxidative stress gene expression between this study and others indicates a multifaceted transcriptional response of oxygen detoxification genes. This response is influenced by divergences in regulator sequences between strains, as in PerR of 630*∆erm* [37], and probably by factors such as length of oxygen exposure and growth phase. It is also conceivable that in *C. difficile *, oxidative stress response genes are sequentially transcribed based on the concentration of oxygen, reminiscent of the expression of iron response genes in *Bacillus subtilis* [46]. *C. difficile* may then optimize its use of particular oxygen detoxification enzymes depending on the exact oxygen microenvironment. Further work is necessary to test this hypothesis.

### Low oxygen triggers changes in metal homeostasis

**Fig. 4.**
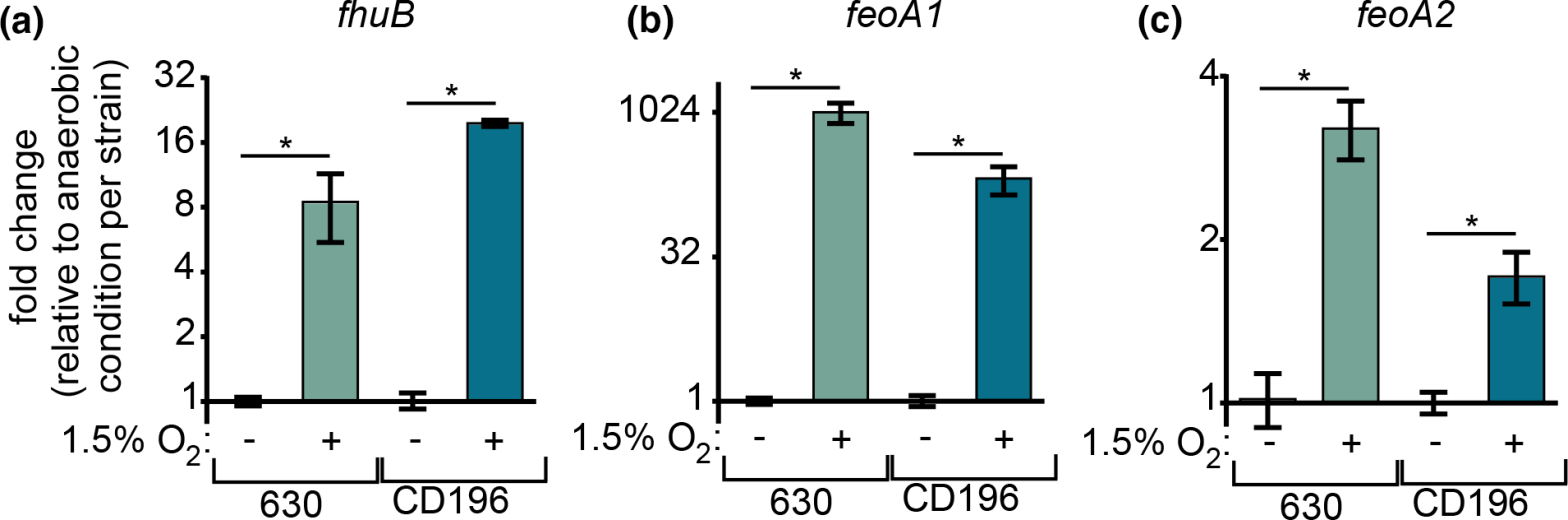
*
C. difficile* upregulates genes involved in metal homeostasis in a low oxygen environment. (**a–c**) qPCR of genes predicted to be involved in siderophore uptake (*fhuB*, **a**) or metal uptake (*feoA1* and *feoA2*, **b, c**). Values shown are transcript fold-change with 1.5% oxygen relative to under anaerobic conditions for each strain. *P<0.05; ns, not significant; two-tailed *t*-test.

One of the largest categories of genes with differential expression in response to 1.5% oxygen were those involved in metal homeostasis (Table S2). These included multiple predicted ferrous iron (Fe) import systems (*feoAB* systems), siderophore uptake (*fhu* operon) and the zinc (Zn) importer *zupT* [47]. qPCR performed on three genes (*fhuB*, *feoA1*, *feoA2*) confirmed significantly higher expression in both strains when oxygen was present (Fig. 4). The most highly upregulated gene in both strain 630 and CD196 was *fldX* (flavodoxin), which probably mediates electron transfer when replacing Fe-containing ferredoxins under Fe-deplete conditions [48]. Overall, the expression pattern of metal homeostasis genes is reminiscent of a metal-starvation response [23, 49, 50]. Many of the genes found in this dataset (*feoA* and *feoB* genes, *fhu*, *zupT*, *fldX*) are known or predicted to be transcriptionally controlled by the ferric uptake regulator (Fur) and repressed in iron-replete environments [48, 49]. When Fe or Zn is limiting, expression of these genes increases and contributes to *C. difficile* survival in the presence of nutrient metal-sequestering host proteins during infection [23, 47]. However, uncontrolled Fe uptake can be damaging, as free Fe reacts with ROS to form toxic hydroxyl radicals via the Fenton reaction [51]. This may explain why metal uptake genes did not exhibit increased expression at high oxygen concentrations in previous studies [17]. At moderate oxygen concentrations (up to 5% oxygen), free intracellular Fe may be detrimental and ultimately toxic to *C. difficile* after prolonged exposure. At lower oxygen concentrations, such as those used in this study, increased metal import could supply necessary cofactors for the Fe–sulphur cluster domains of Rbrs [41], which may mitigate oxidative damage when oxygen is present at approximately 1.5% in the environment. An alternative explanation is that soluble ferrous Fe in the medium is oxidized to ferric Fe by oxygen, essentially starving *C. difficile* of bioavailable Fe. While the RNA-seq data in the present study do not delineate between the mechanisms by which oxygen triggers transcriptional changes in metal homeostasis genes, adaptation to microaerobiosis may involve direct oxygen sensing or damage as well as a response to oxygen-dependent changes in nutrient availability.

**Fig. 5.**
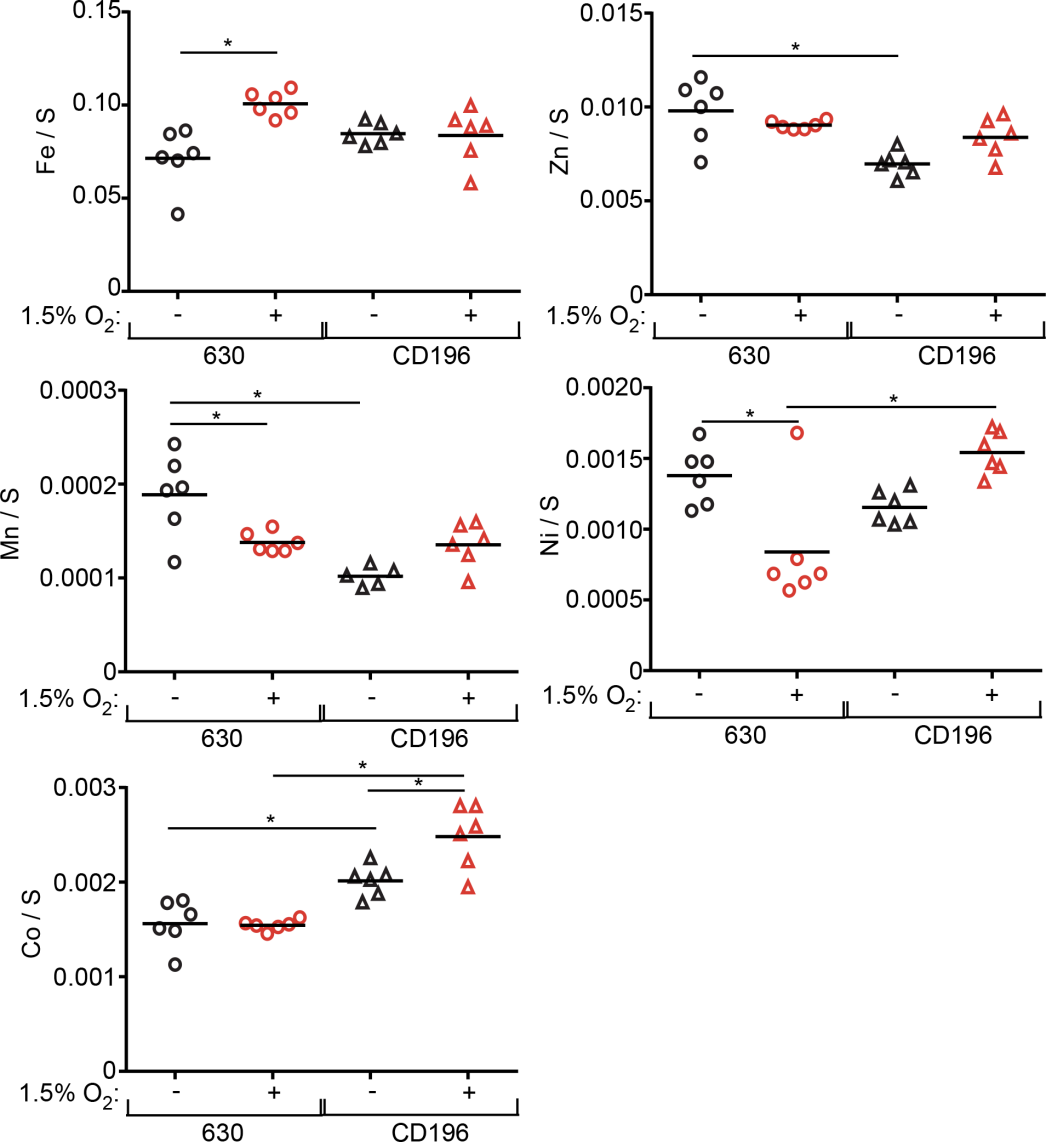
*
C. difficile* strains exhibit differences in intracellular metal under anaerobic and aerobic conditions. Concentrations of intracellular metals normalized to sulphur concentrations for each strain under anaerobic or aerobic conditions. Each value represents an independent sample. Isotope numbers are as follows: ^34^S, ^56^Fe, ^66^Zn, ^55^Mn, ^60^Ni, ^59^Co ; *P<0.05, two-way ANOVA followed by Sidak correction for multiple comparisons.

Based on the high level of expression in the RNA-seq dataset of transcripts involved in metal uptake, we predicted that an oxygenated environment would alter intracellular metal levels. To test this hypothesis, metal concentrations were assessed from both strains using ICP-MS when grown either anaerobically or with 1.5% oxygen (Fig. 5). In a microaerobic environment, strain 630 contained higher concentrations of Fe, but lower concentrations of manganese (Mn) and nickel (Ni). This pattern did not hold true for CD196, which only had a significantly higher concentration of cobalt (Co) with oxygen. These results are somewhat surprising, as the similar expression pattern of metal-responsive genes between the two strains did not correlate with similar changes in intracellular metal levels. One possible explanation lies in the multiple *feoA* and *feoB* annotated genes in *C. difficile *, as both *C. difficile* strains encode three *feoB* genes and five *feoA* genes. To ease direct comparison of *feo* genes between strains 630 and CD196, genes with identical sequences were given equivalent annotations (Fig. S4). In strain 630, under microaerobic conditions, there is more than 3-fold higher expression of *feoB1*, *feoA1*, *feoA2*, *feoA3* and *feoA5*. Under the same conditions, CD196 also increases expression of *feoB1*, *feoA1* and *feoA3*; however, in contrast to strain 630, CD196 expression of *feoA4* is also increased more than 3-fold while *feoA5* is not (Table S2). The consequences of the divergent *feoA* gene expression patterns is unclear, but they may be functionally relevant. Together, *feoA* and *feoB* import ferrous Fe, though the mechanism of how these proteins interact to transport Fe remains unknown [52, 53]. Thus, while they are predicted to import Fe, there may be differences in their metal selectivity or the import rate stemming from interactions between the multiple FeoA and FeoB proteins. Indeed, in *Porphorymonas gingivalis* FeoB was shown to import Mn [54], providing evidence for divergence from canonical ferrous Fe import. Further work is required to characterize the metal specificity and regulation of the multiple *feo* genes in *C. difficile *.

### *C. difficile* strains 630 and CD196 express genes in a distinct manner that is both dependent and independent of oxygen

*C. difficile* strains are known to have diverse phenotypes both in culture and during infection. These strain-specific properties can be driven by a variety of factors, including altered genomic content [55] or variable gene expression patterns [56, 57]. To address the latter possibility, the transcriptomes of *C. difficile* 630 and CD196 were directly compared under anaerobic and microaerobic conditions. This strategy required the creation of a shared genome to allow for cross-comparison of RNA-seq results from both strains. To generate this resource, a consensus sequence for every shared gene (≥80% similarity) was created via blast to compare each gene from *C. difficile* 630 to each gene encoded in the CD196 genome. Individual consensus sequences were then collected (referred to as the ‘consensus genome’) and served as the basis for further analysis (Table S3).

**Fig. 6.**
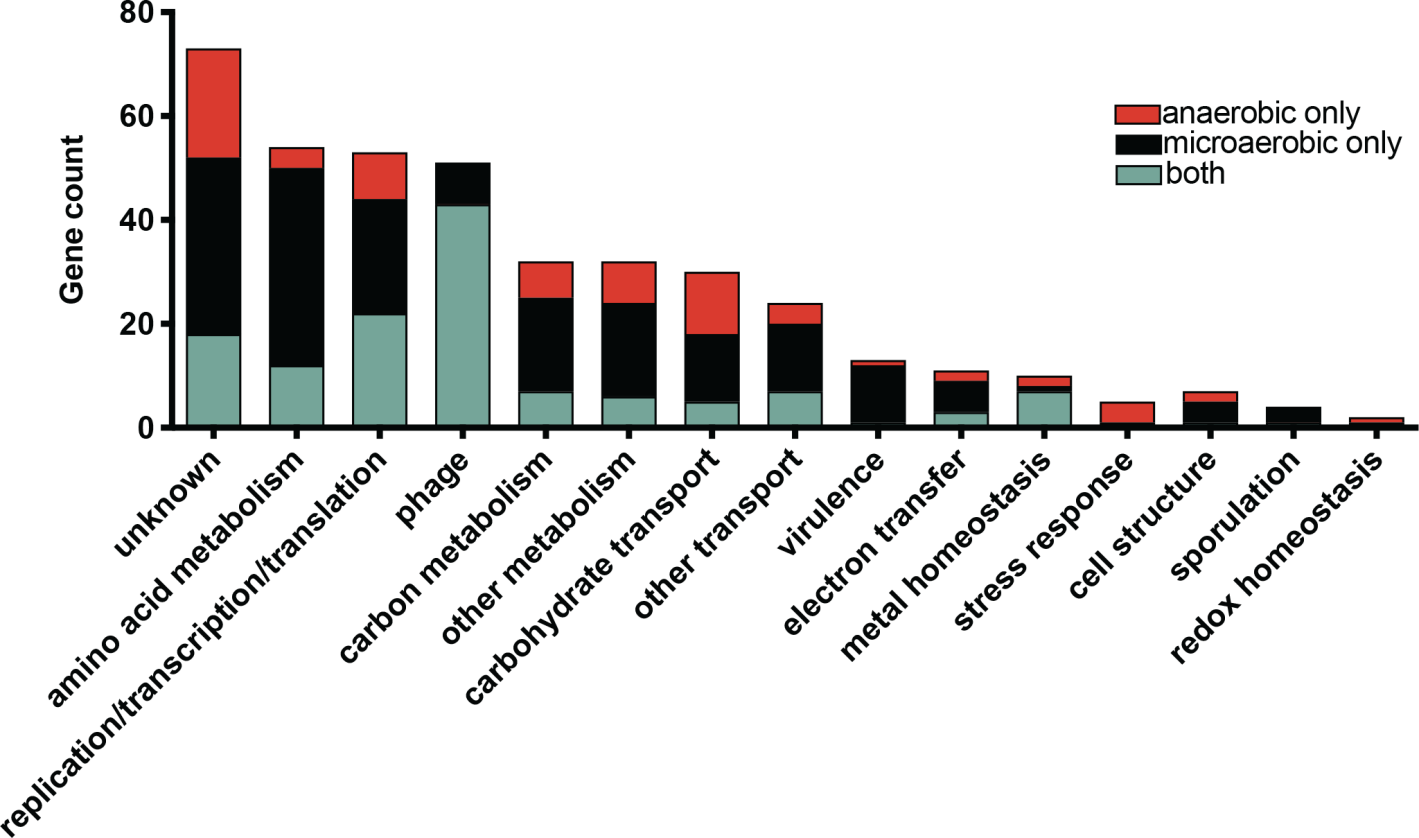
A transcriptional comparison of *C. difficile* strains 630 and CD196 reveals differences in basal and adaptive expression patterns. Comparison of gene expression under anaerobic and aerobic conditions for both strains. In total, 130 genes showed at least a 3-fold difference in expression (positive or negative) between the two strains, independent of the condition. Eighty genes were differentially expressed only under anaerobic conditions, while 192 genes displayed differences in expression only under microaerobic conditions. Genes are grouped into functional categories to identify pathways and cellular processes that are differentially expressed in the tested conditions.

Between strains 630 and CD196, 80 genes were differentially expressed only under anaerobic conditions while 192 genes were differentially expressed only under microaerobic conditions (Tables S4 and S5, Fig. 6). In total, 130 genes displayed differential expression under both conditions. In general, the genes with differences in expression fell into multiple categories including prophage genes, amino acid metabolism, carbohydrate metabolism, metal homeostasis and nutrient transport (Fig. 6, Table S5). These changes offer insight into how conserved genes can be alternatively expressed by different strains either at a basal level or in response to environmental stimuli, such as the presence of oxygen. Specifically, the cross-comparison revealed subsets of genes that (i) are differentially expressed anaerobically but reach similar expression levels with oxygen; (ii) aid in the adaptation to a microaerobic environment in a strain-dependent manner (microaerobic only); and (iii) are differentially expressed independent of the tested environmental oxygen condition (both anaerobic and microaerobic).

One of the largest categories of differentially expressed genes under both microaerobic and anaerobic conditions were prophage genes (Fig. 6). To simplify comparisons, annotations are shown using the *C. difficile* 630 gene nomenclature, with all genes described in this section having homologues in CD196 based on DNA sequence identity (Table S3). Prophage genes encompassed three main gene clusters: *CD630_09450 – CD630_09680*, *CD630_13620 – CD630_13730* and *CD630_28990 – CD630_29230*. Based on our RNA-seq data, these prophage genes remained integrated in the bacterial chromosome and are expressed at a low level, rather than being excised (Table S1). Prophage genes can influence several regulatory pathways relevant to CDI, including toxin expression [58]. No annotated regulatory or virulence genes are encoded within these three prophage regions, and thus the relevance of their relative abundance is not known.

Several genes encoding sugar transporter or carbon metabolism enzymes varied between the two *C. difficile* strains. Under anaerobic conditions, CD196 had higher expression of two operons encoding a fructose/mannitol-family phosphotransferase system (PTS) transporter (*CD630_25550–25560*) and fructose-specific PTS transporter (*fruABC*), but lower expression of a mannose-specific transporter (*CD630_30130–30160*). Under microaerobic conditions, there were even more striking differences in transcription of sugar transporters. Four gene clusters involved in sucrose transport (*CD630_04690*), mannose transport (*CD630_25670–25680*), lactose/cellobiose transport (*CD630_34430–34450*) and xylose utilization (*xylA*, *xynA*, *xynB*) exhibited higher expression in strain 630 than CD196. *C. difficile* strains are known to exhibit variability in sugar fermentation, particularly for sugars aside from glucose and fructose [59]. These data support the notion that these strain-dependent differences in sugar utilization could be due to variation in the expression of multiple PTS transporters, which is influenced by environmental conditions.

A key difference in expression between strains 630 and CD196 involved genes for amino acid metabolism, particularly under microaerobic conditions (Fig. 6). Genes encoding enzymes involved in the biosynthesis of aromatic amino acids (*aro* operon) and histidine (*his* operon) were more highly expressed in CD196 relative to 630. While the fate of the synthesized amino acids remains unclear, aromatic amino acids (phenylalanine, tryptophan and tyrosine) can serve as electron donors for Stickland fermentation [60]. Coupled to amino acid oxidation, *C. difficile* can reduce glycine and proline [44, 61]. In the presence of oxygen both *C. difficile* strains increased expression of the glycine reductase operon (*grd*) (Fig. S3), which may indicate increased reliance on Stickland fermentation.

**Fig. 7.**
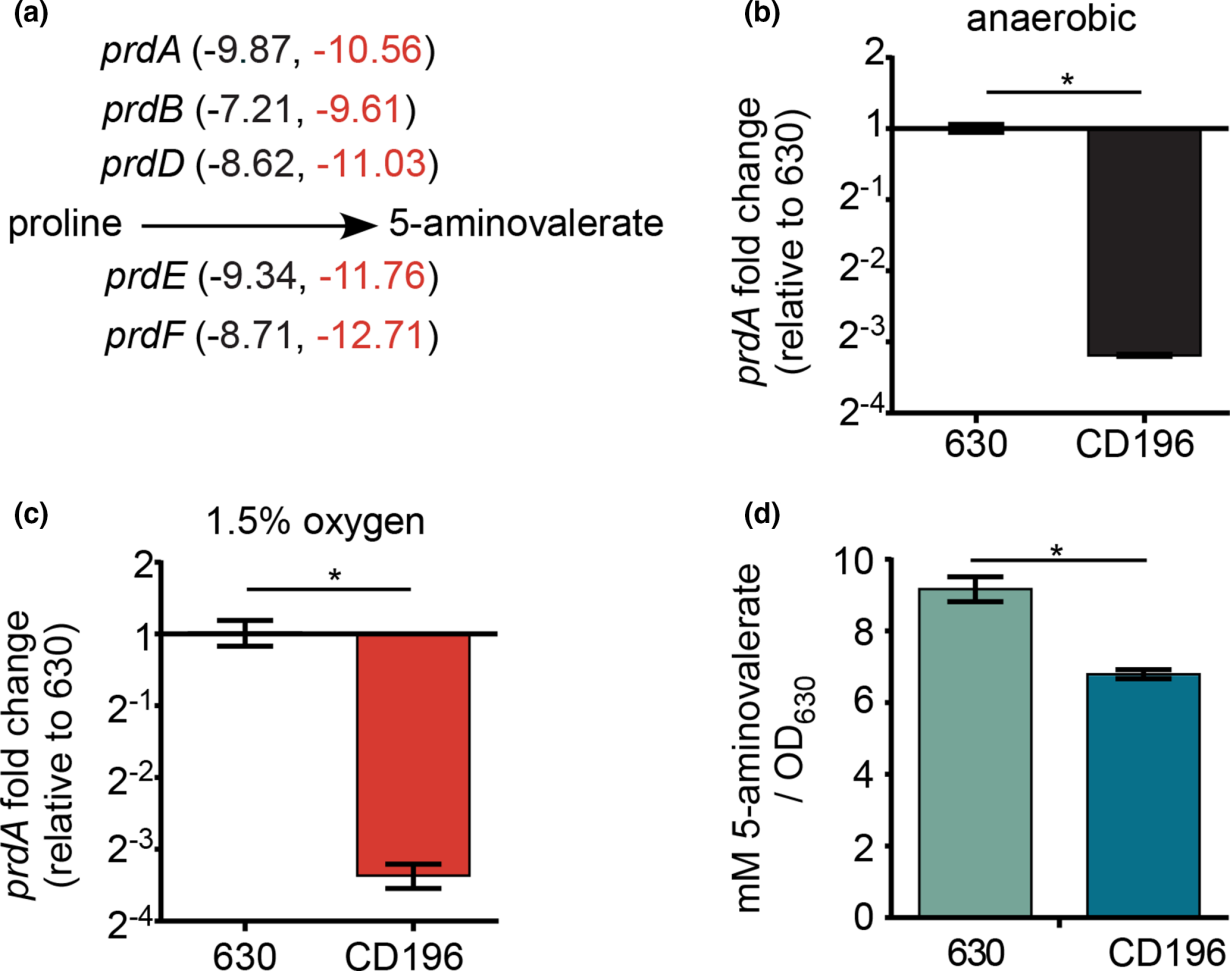
*
C. difficile* strains show differences in proline fermentation independent of oxygen. (**a**) Proline reduction pathway with genes encoding the proline reductase (*prdABDE*), proline racemase (*prdF*) and regulator (*prdR*). Values are from the RNA-seq experiment and represent relative fold-change of *C. difficile* strain CD196 compared to 630 under anaerobic conditions (left value) or with 1.5% oxygen (right value). (**b, c**) Quantification of *prdA* transcripts via qPCR relative to *C. difficile* strain 630 under anaerobic conditions (**b**) or with 1.5% oxygen (**c**). (**d**) Normalized concentrations of 5-aminovalerate under anaerobic conditions taken at early exponential growth. *P<0.05, two-tailed *t*-test.

To further ascertain how differences in gene expression could alter fundamental aspects of *C. difficile* metabolism, we focused on proline fermentation. *C. difficile* can ferment the amino acid proline into 5-aminovalerate via Stickland fermentation, which provides the bacterium with a means to maintain redox balance and produce ATP [44]. *C. difficile* ferments proline *in vivo* [23], and changes in proline concentrations in humans are associated with *C. difficile* colonization [62]. In our cross-comparison (Fig. 6), the genes encoding the proline reductase (*prdABDE*), proline racemase (*prdF*) and the *prd* regulator (*prdR*) were expressed at significantly lower levels in CD196 than in 630 under both anaerobic and microaerobic conditions (Fig. 7a). This pattern was validated through *prdA* expression detected using qPCR (Fig. 7b, c). To determine if the difference in gene expression altered proline fermentation, 5-aminovalerate was measured from spent media of strains 630 and CD196 cultured anaerobically. In agreement with the expression data, significantly lower levels of 5-aminovalerate were measured in the spent media from CD196 compared to 630 (Fig. 7d). Overall, our cross-comparison supports the notion that there are basal strain-dependent differences in *C. difficile* gene expression that could have crucial consequences in the metabolism, growth and virulence of *C. difficile *.

The present study has limitations. While 1.5% oxygen is a reasonable estimation of a physiologically relevant oxygen concentration in the intestines that permits *C. difficile* growth, oxygen in the gut fluctuates from fully anaerobic to levels higher than tolerable by *C. difficile* [63]. Additionally, the direct effects of oxygen on *C. difficile* were not differentiated from oxygen-induced changes in nutrient availability, such as Fe oxidation. Nevertheless, though the mechanisms of oxygen sensing remain unclear, our data describe *C. difficile* transcriptional responses to long-term growth in a microaerobic environment. For strain comparison of transcript abundance, our analysis relied on creating a consensus genome where a gene in CD196 could be directly compared to a homologous gene in 630. The corresponding genes were determined through blast with stringent cutoffs (see Methods) and we are confident that this approach accurately compared homologous genes. Still, there is a possibility that homologous genes were missed, particularly for short genes or those with significant sequence divergence. Lastly, the transcriptomic differences observed on the basis on our consensus genome offer insight into how both strains use their shared genomic content to adapt to changing oxygen pressures; however, the non-consensus genome (Table S3) probably contributes to the adaptation to dynamic environments, a hypothesis that is supported by the exceptional genomic plasticity of *C. difficile* and the contribution of newly acquired traits to its lifestyle [2]. Further studies will be necessary to elucidate how genomic differences between 630 and CD196 influence *C. difficile* physiology directly (e.g. through novel traits) and potentially account for the transcriptional differences observed in the consensus genome.

## Conclusions

Oxygen is a significant contributor to structuring the ecology of microbes, whether free-living or associated with a host. This is in part due to the toxic effects of ROS created spontaneously, via metabolic processes, or as part of a host immune defence. To survive in an oxygenated environment, organisms have evolved oxygen-resistant metabolic pathways and ROS detoxification systems. While *C. difficile* is considered an obligate anaerobe, we demonstrate here that *C. difficile* can adapt to grow in an environment with physiologically relevant levels of low oxygen. This adaptation includes increased expression of genes encoding oxygen detoxification enzymes and metal homeostasis proteins, along with altered expression of genes for energy-producing pathways. Additionally, using our comparative genomic approaches we observed significant oxygen-dependent and oxygen-independent differences between *C. difficile* strains 630 and CD196, suggesting that as a species *C. difficile* probably varies in its ability to grow in an oxygenated gut. Overall, these data indicate that *C. difficile* is well suited to survive multiple environmental stresses, though responses may be strain-dependent and probably contribute to the highly variable clinical manifestations of colonization and disease.

## Supplementary Data

Supplementary material 1

Supplementary material 2

Supplementary material 3

Supplementary material 4

Supplementary material 5

Supplementary material 6
