# Supplementary material for: Clostridioides difficile strain-dependent and strain-independent adaptations to a microaerobic environment
Source: Microb Genom. 2021 Dec 15;7(12):000738. doi: 10.1099/mgen.0.000738 (PMC8767335; doi:10.1099/mgen.0.000738)
Supplement: Supplementary material 1 [file mgen-7-0738-s001.pdf]

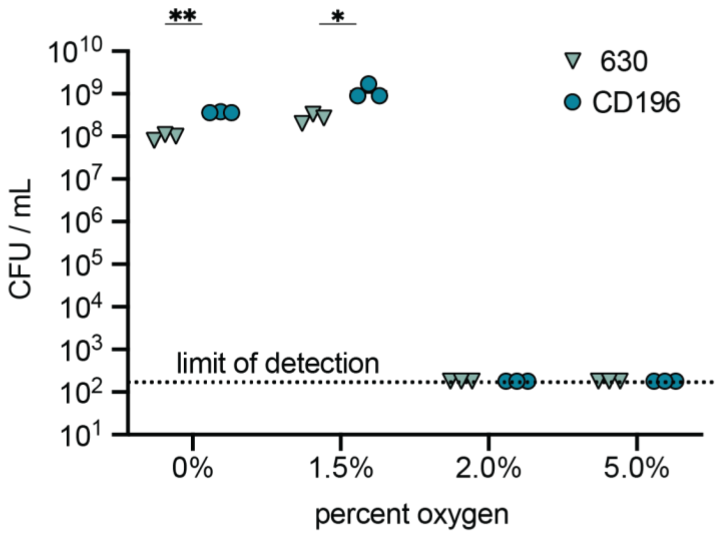

Supplemental Figure 1: *C. difficile* density in culture after 16 hours of growth. Both *C. difficile* strains 630 and CD196 were grown in BHIS broth (without cysteine supplementation) for 16 hours at 37 °C under 0%, 1.5%, 2%, and 5% oxygen. Vegetative cells were enumerated by plating on BHIS agar. Unpaired *t*-test corrected for multiple comparisons; * = $p<0.05$; ** = $p < 0.01$

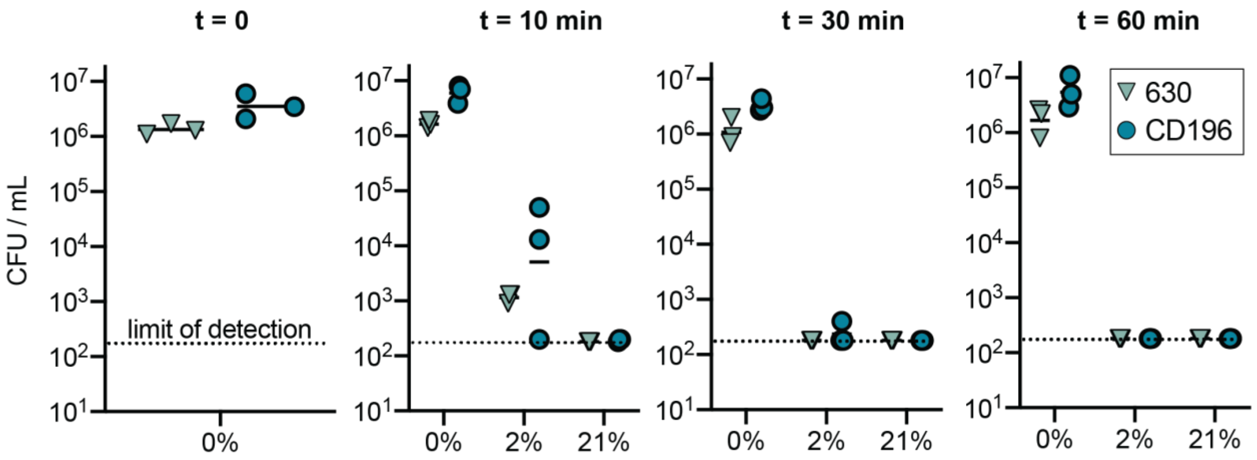

Supplemental Figure 2: *C. difficile* survival after exposure to oxygen in culture. *C. difficile* strains 630 and CD196 were grown in BHIS broth (without cysteine supplementation) for up to 60 minutes following exposure to 0%, 2%, or 21% (atmospheric) oxygen. Vegetative *C. difficile* cells were enumerated by plating on BHIS agar.

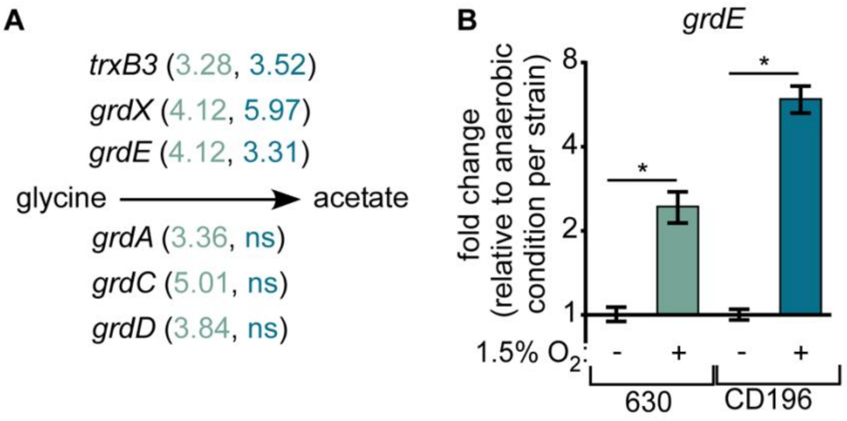

Supplemental Figure 3: Low oxygen alters expression of glycine reductase genes. (A) Glycine reduction pathway with genes encoding the glycine reductase. Values are from the RNA-sequencing experiment and represent relative fold-change of *C. difficile* cultured with 1.5% oxygen compared to anaerobically for strain 630 (green) and CD196 (blue). **(B)** Quantification of *grdE* transcripts via qPCR expressed with 1.5% oxygen compared to under anaerobic conditions. * = p<0.05, two-tail t-test

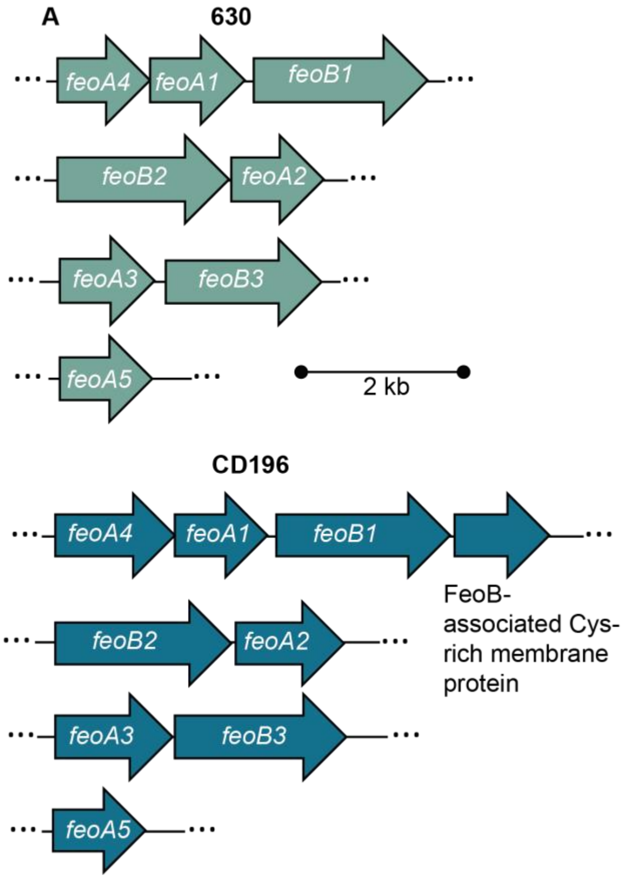

B

Gene ID	Gene name	Location
CD630_14790	*feoB1*	1,714,822..1,716,978
CD630_15170	*feoB2*	1,758,443..1,760,563
CD630_32740	*feoB3*	3,831,876..3,833,822
CD630_14780	*feoA1*	1,714,501..1,714,722
CD630_15180	*feoA2*	1,760,589..1,760,816
CD630_32730	*feoA3*	3,831,640..3,831,858
CD630_14770	*feoA4*	1,714,276..1714,488
CD630_17451	*feoA5*	2,022,187..2,022,411

Gene ID	Gene name	Location
CD196_RS07420	*feoB1*	1,573,966..1,576,122
CD196_RS07620	*feoB2*	1,617,342..1,619,462
CD196_RS16610	*feoB3*	3,661,039..3,662,985
CD196_RS07415	*feoA1*	1,573,645..1,573,866
CD196_RS07625	*feoA2*	1,619,488..1,619,715
CD196_RS16605	*feoA3*	3,660,803..3,661,021
CD196_RS07410	*feoA4*	1,573,420..1,573,632
CD196_RS09130	*feoA5*	1,931,410..1,931,634

Supplemental Figure 4: *feoA* and *feoB* genes in *C. difficile* 630 and CD196 (A) Arrows represent distinct *feoA* and *feoB* genes in both *C. difficile* strains. Homologous genes are similarly annotated. **(B)** Gene ID and chromosomal location of each *feoA* and *feoB* gene. Gene annotations are those used in this study.
